# Supplementary material for: Patterns of Intron Gain and Loss in Fungi
Source: PLoS Biol. 2004 Nov 30;2(12):e422. doi: 10.1371/journal.pbio.0020422 (PMC532390; doi:10.1371/journal.pbio.0020422)
Supplement: Table S1 — Also available at http://genes.mit.edu/NielsenEtAl/. (4.3 MB ZIP). [file pbio.0020422.st001.zip › NielsenEtAl/html/694.html]

AN4413.1.NCU01486.1.MG07174.1.FG04361.1


```
 CLUSTAL W (1.82) Multiple Sequence Alignments - Introns Inserted


Sequence 1: MG07174.1	1305 aa
Sequence 2: FG04361.1	1297 aa
Sequence 3: NCU01486.1	1342 aa
Sequence 4: AN4413.1	1357 aa
Alignment Length: 1446 aa
Number Identitical Residues: 433 aa
Alignment Score (without introns) 23537


MG07174.1 	------------------------------------------------------------
NCU01486.1	------------------------------------------------------------
FG04361.1 	------------------------------------------------------------
AN4413.1  	MNAATALRARMATSFVARRGFSTTRSQLSSPYHYAEGPRSNIPFNPLTKYFFFRYWAFMI
          	 .::::  :  :::  :  . ::: :. ::.   :... :. . .. :.       :   

MG07174.1 	~-----------~MRNLRNIRYDVCKTD-------VDVTA~TCWDAGSDDILVTFGPTEA
NCU01486.1	~-----------~MRNLRNIGHGAYRPGQEQPLPSNSISA~SCWDVSRDEIILAHGPTQD
FG04361.1 	~-----------~MRNLRNIRFGQFQR--------ANVTA~ACWDPEKDEVLCVAGPTEQ
AN4413.1  	1TGFGAPFAIAA1FSSLANLQDPLNPPPR---APGVSGNA1TAWDTASDAVICTFGPTPT
          	 :. .:. : :: : .* *:             .. . .* :.**   * :: . ***  

MG07174.1 	DPSITLARISDEP----GS~SRL2KCVTVASWEAP----SPHPDIPFDQVVSLHHFSDNA
NCU01486.1	FSRVELVRLVKDSS---EP~SNL2ECKLVASWDAAPVLRQNGSEPEPDTIKSLHYFPDTL
FG04361.1 	SSGIELLRVAGDQ----DI2S--~-VRTVATWDAP----SPTPDLAVDRVVSLQYLSGSA
AN4413.1  	NPVIELRRKRADVYFSDAV~SPD~VFDTIASWDAP----CPLPHLDCDHVLSLHYFADTL
          	 . : * *   :   :.   *       :*:*:*.       ..   * : **:::... 

MG07174.1 	TTCLVLAGGDVVTVQEDQHATAPGTAHIEIAGSIDAGIAAARWAYDDELLVLATKVDTVI
NCU01486.1	TTCVIMAGGDIITVVEDDY-GVPGEAHVEIVGTLEPSVAGARWSPDEELLIVATGDAKVL
FG04361.1 	TTCLVLEGGDIITVQEDEFSGQD--AHIEIVGSIDAGIAAARWSPDEELLIVVTKENNVI
AN4413.1  	TACLVLEGGDIIVVREEPLPGED---KIEIVGSVDVGITAAAWSPDEELLALTTSAHTFL
          	*:*::: ***::.* *:  .      ::**.*::: .::.* *: *:*** :.*   ..:

MG07174.1 	FMSRSFDAIAEVTMTVDDLQASKHVSVGWGKKETQFQGKG--AKALRDPTIPEKVDRGLP
NCU01486.1	FMSRTFDVITSATMTEDDLKLSKHVSVGWGKKETQFQGRGAKAKALRDPTIPEKVDEGRL
FG04361.1 	FMGSTFDPVAEITMTVEDLNASKHVSVGWGKKETQFQGRG--AKAMRDPTIPEKVDEGVP
AN4413.1  	YMTREFENVAEITFTPDDLKASQHVSVGWGKRETQFQGKR--AKAMRDPTVPEKVDEGKL
          	:*   *: ::. *:* :**: *:********:******:   ***:****:*****.*  

MG07174.1 	SALED-SSTTISWREDGAYVAIN--SVRAGSRRVIRVYTREGVLDSASEPVDCLEGSMSW
NCU01486.1	SANDDLHKCSISWRGDGAYVAVNFFSQESGGRRVIRVYNRDGELDSVSEPVDGLEGSLSW
FG04361.1 	SPQED-GATTISWRGDGAYVAIN--SVQEGSRRVIRVYSREGELDSASEPVDGFESALSW
AN4413.1  	SGNDD-GRTTITWRGDGAFVAVN--SIVEGIRRAIRVYSREGTLDSVSEPVDGLEGALSW
          	*  :*    :*:** ***:**:*  *   * **.****.*:* ***.***** :*.::**

MG07174.1 	RPSGNLIAGIQRLSDRVDVVFFERNGLRHGQFTLRFPPD-LLSTNIPINLEWNPDSTVLA
NCU01486.1	RPEGNLMAGIQRFSDHVDVVFFERNGLRHGQFTLQIPQD-RPEVAEDLALEWNSDSNVLA
FG04361.1 	RPAGNLMAGVQRFSNRIDVVFFERNGLRHGEFTLRSPSG-PVEAHEKIHLEWNSDSTVVA
AN4413.1  	RPYGNLIAGIQRLDDRIDVVFFERNGLRHGQFTLRLTEEERSTWASNIHLSWNVDSTVLA
          	** ***:**:**:.:::*************:***: .  .       : *.** **.*:*

MG07174.1 	VSFGGTVQLWAMGNYHWYLKQEIRCASTCFRP--------------SWHPEKPMRLSLVC
NCU01486.1	VIMRDRVQLYTMGNYHWYLKQELPCAGYAQLASQFQGESRWTLPWFSWHAEKPLLFAVAA
FG04361.1 	VIFKDMIQLWTMGNYHWYLKQEMPIEANSTCL--------------SWHPEKALRFAAAS
AN4413.1  	VQFKDRIQFWTSGNYHYYLKQEIPVIVSSEGP-----------FAYKWHHEKALRFVAGA
          	* : . :*::: ****:*****:     .                 .** **.: :   .

MG07174.1 	KN1SVAHTEWVFHTSRGSILPPHDYGAVAVIDGN~NIKITPFRTATIPPPMAMFELEAAT
NCU01486.1	AE1RAVWFEFVLAIARGPMYG--GMGDVAVIDGR~TIKFTPFQTANVPPPMALYDIEVDY
FG04361.1 	TS~NVVVAEHIFYTARGSCLPPHDNGAVAVIDGE~TVKLTPFRTVNVPPPMSMFDITASA
AN4413.1  	SE1SILDGEFVFKVFHGSTTPPNDVGAVAVIDGK1TLKLTPLKLSGVPPPMAHNELPLDA
          	 .      * ::   :*.   . . * ******. .:*:**::   :****:  ::    

MG07174.1 	CVIDVAFTPNNSHMAVLHRTGVDIYEWQTK--AGRVLCPKRTINVPLKAAGPHDTTSPLQ
NCU01486.1	PVTDIAFSKDASQMAVLHQKGMHLFALEKQGPAGRRAAPKLIKTISLDNFEN---KCQLQ
FG04361.1 	AVVDVAFGRDNTSFAILHRKGIDIYTWPVK--NGRPIKPQVSKKITFDEMASPGYNVLLR
AN4413.1  	NAIDVAFSKSGTRIAVLMNDHFSVYLWSLK--SRPVPVPILESSYPLSGEIG---SRPRQ
          	 . *:**  . : :*:* .  . ::    :        *    . .:.       .   :

MG07174.1 	VAMSSPEDLALLSVK-SNKPFASIYKLASGKPELLCELDAQG---FSGFVFSATAPILPC
NCU01486.1	IAFTAPSQVQVLSLD-DFQ--LQITAWDFNEELMLGEVGTGLQAVTLTSADETSVEDGTV
FG04361.1 	IAAVA-DAFHYFGFE-EEKGFVQRSVQAIGEGETSAAVINSQEVLVATASYQDDSSFAGY
AN4413.1  	ITFLNDNEVYILRSRGPNNTCIERTTLETRETKVVYEAADSEHLLTMFSSLGHEALWFSH
          	::    . .  :   .  :   .       :                             

MG07174.1 	VQDRSGKLLQPSAEGQALLPVKFSTQLPWAEVILHDDSTLAVGMSRGGQLYANTRLLAKN
NCU01486.1	VQDRQGNITRISVENGETVLGKFPTLLPWATYTTYEDQFIAFGLSRNGHLYANSRQLVKN
FG04361.1 	GQDNSGKLFYIGESGNEILPVQFQTQLPWFEISKVDDEIVAFGLSRNGHIYANSRLLAKN
AN4413.1  	VPRPGQSIAYSTIT---MPSSNESQVTPWAQSPVAD------------TYWAKSAQISDD
          	      .:             :     **      :              :*::  : .:

MG07174.1 	CTSFVVTGDHLIFTTTNHFLK~LVHLTG-VEE1LEVPADDPEVDERCRSIERGARLVTAV
NCU01486.1	CTSFVVTDEHLIYTTSNHFVK~FIHLTANVEE~LDVPLDDPETDERCRSIERGGRLVTAM
FG04361.1 	CTSFVVTPSHLIFTTNNHLVK~FVHLSANIDE1LEVPEDDPETDERCRSVERGSRLVTAI
AN4413.1  	--------------------E0FVHLRR-AED1MEAPPDTPETDERCRSIERGSRLVTVM
          	                    : ::**    :: ::.* * **.******:***.****.:

MG07174.1 	PSNMNVVLQMPRGNLETIFPRAMVVAGIRKLINDKNYARAFSYCRTQRVDMNILYDHRPE
NCU01486.1	PSRMSIVLQMPRGNLETIYPRAMVLAGIRQLVEQKEYGAAFTTCRTQRVDMNLLYDHRPE
FG04361.1 	PSNMSIVLQMPRGNLETVFPRALVVAGIRNLIDEKNYARAFSYCRTQRVDMNILYDHQPE
AN4413.1  	PSAFAVVLQAPRGNIETIYPRALVLAGIRSFIDKKDYRSAFLTCRSQMVDMNLIHDYAPE
          	** : :*** ****:**::***:*:****.:::.*:*  **  **:* ****:::*: **

MG07174.1 	QFLANVGLFLDQLGDTSYIDLVLSSLR2EEDVTQTMYKDTKKSKPAWAPSSTPLPSQENP
NCU01486.1	QFLENVGLFLDQVKNAADIDLFLSTLK~EEDVTQTMYRNTKTGV-----VATPIQPALIT
FG04361.1 	QFLANVGLFLDQIPDVAHIDLFLSSLR2VEDVTQTMYQDTKRSK---AFGADAVASDLSP
AN4413.1  	QFMESITLFVDQVKRVDFVDEFLSRLK2EEDVSQTLYKDTLKALD----MEVAAETGFTM
          	**: .: **:**:  .  :* .** *:  ***:**:*::*  .         .  .    

MG07174.1 	PSVSLSDPSSSKVNTVCDAVLKELEARNPASLQNIITAHVCKNPPALDDGLSVIAGLMK~
NCU01486.1	APK------TSKINTICDAVLHSLKAKKNANLQNIITAHVCKNPPALSDGLQVVASLME~
FG04361.1 	APR----GSVAKVNTVCDTLLGALQSRKATNLQNTITAHVCKSPPALDDGLLLVSELMQ~
AN4413.1  	TGK-----KGSKVNRICDGFLTALEKRSDTNLHNLITAHVCKLPPDLESGLQLVARLRE1
          	.       . :*:* :** .*  *: :. :.*:* ******* ** *..** ::: * : 

MG07174.1 	ENEALAEKAVEHICFLVDVNRLYDNALGLYNLELALLVAQQSQR~DPREYLPFVQSLHQL
NCU01486.1	EDESLAERAVEHICFLVDINKLYDHALSLYNLELTLLVAQQSQR~DPREYLPFIQSLHKM
FG04361.1 	EDEKIAEKAVEHICFLVDVNRLYENALGLYNLELALLVAQQSQR~DPREYLPFIQNLHSL
AN4413.1  	ESPEQAEDAVEHMCFLTDANRLYDTALGLYDLELTLLVAQQAQR0DPREYLPFLRKLQQL
          	*.   ** ****:***.* *:**: **.**:***:******:** ********::.*:.:

MG07174.1 	SQLRRCFTIDDHLGNREKALGHLKADS--DFEGVKDYAVKHSLYQDALGLYRYEAEQSRT
NCU01486.1	DPLRRQFTIDDHLSFHEKALVHLRAIANTYSDEVESYIVKHQLYPSALALYRNEPTPLRT
FG04361.1 	TELRRKFEIDDHLERRVKALGHLQTMD--VFDELLAYTTKHSLYHDSLRLYRYDPPRLRE
AN4413.1  	PDLRRFFEIDNYLGRWQKALGHLHGLH--AHDELREYVVKHVLYKDAIDIYKYEPEQLRD
          	  *** * **::*    *** **:       : :  * .** ** .:: :*: :.   * 

MG07174.1 	LMALYAEYLESNSRNREAGLA~YEALGKFAEAMSCYRAAGPSSWRECLAAAQKQQPPPTK
NCU01486.1	ITALYAAHLRSLSKFRDAGLA~YESLGDYPAATECYLKAGTSSWRECLFTSS-LDPSLTP
FG04361.1 	LTAAYAAYLESTSAYREAGLA~YESLENYAKATSCYRTAGATCWQECLYTAAQQQPPMST
AN4413.1  	ITHLYADHLYQESQYKDAGIA1YESLSMYTDAYKCYQLA--HLWRESLYTAM--LVPLSQ
          	:   ** :* . *  ::**:* **:*  :. * .**  *    *:*.* ::     . : 

MG07174.1 	SSLTELAESLADALN-EAKDYVGAATIQRDFLNDPATAIQLLCKGSLFADAMLLAATSSE
NCU01486.1	DQRHEIASTLADALR-EAKDWAAVATIQAEHLASLPSAISALCKGYLFADAFRLISLHSR
FG04361.1 	ESMADLASNLADALW-EAKDYSSAATIHLEYLDSIDMAVSCLCKGYHFADAIRLVVQHNR
AN4413.1  	DELTTHATDLATTLVEENKDYLAASQIHAEHLHDIPTAARLLCRGARYSEATRLLTLHSV
          	..    *  ** :* .* **: ..: *: :.* .   *   **:*  :::*  *    . 

MG07174.1 	AG-LDGEVDSGLTEAFASSTELLADCKAQLKAQTPRILDLRRKAAEDPLGFYEGE-RPGG
NCU01486.1	PELLESHLDPGLLDAFSSSTEFLADCKSQLKAQVPRIAELRLKAAEDPLAFYEGE-NPFG
FG04361.1 	PDLLTTSVDTGLADALGTTTEFLADCKAQLKAQIPRVAELRRKAIEDPLAFYEGD-RAGG
AN4413.1  	QSLIPEIVDVALADAMGSMTDLLADFRSQLQAQVPRIAELRVRRIQDPLAYFGGDPTATD
          	   :   :* .* :*:.: *::*** ::**:** **: :** :  :***.:: *:. . .

MG07174.1 	G--DGDMPDDVSVAASSRLSTS-ASLFTRYTGKEG-SIGTAGTGVSRATHKNRKREEKKR
NCU01486.1	ARTGGDIPDDISVAASSRVSTS-ASLFTRYTGKGSQVTGTVASNVSRATSKNRKREEKKR
FG04361.1 	----MDIPDDVSVAASSRVSTS-ASLFTRYTGKAG-SVGTAGTGVSRATSKNRKREEKKR
AN4413.1  	GAAGVDIPDNVSLAATDASTLAGKSMFTRYTGKTS----SGKTTSSRQSSRNRRKEERKR
          	. :. *:**::*:**:.  : :. *:******* .    :  :  ** : :**::**:**

MG07174.1 	ARGRKGTVYEEEYLVNSTRRLIERVN-SVGGETEALVAGLFRRGMLEQARAVEALLAEVV
NCU01486.1	ARGRKGTVYEEEYLVNSVRRLVERVEGSARLEVERLVCALVRRGMSERARAVEGLMAEVV
FG04361.1 	ARGRKGTVYEEEYLVNSIRRLIDRVS-AAAPDTERLIFALVRRNMPERARAAESLMAEVS
AN4413.1  	ARGKKGTVYEEEYLVNSVRRLIERVG-TTVPEVENLVDSLLRRGMRERAAAIEKATRDVL
          	***:************* ***::**  :.  :.* *: .*.**.* *:* * *    :* 

MG07174.1 	EGCRLAVQQVWPGSE----AKTADGQQ---------SGQAATGGDAVLEAAMEARV---A
NCU01486.1	EGCLRAVEEVWPQQQQQ--QQQQEGGGEQDASGWEGAEWRPTGGDAVLADSIEAMR---R
FG04361.1 	DACTSAVSEVFKSSEPE--GEKKNEGE---------PAWHATGGEAVLQDFVMGQG---K
AN4413.1  	KLCEESQEEVFIVQQKQQLEEGENGEQGGEAVNGPVDVPMPQGGQRVFWESIVSTLGGGK
          	. *  : .:*:  .: ..  :  :     .: .       . **: *:   : .  ... 

MG07174.1 	KQEPPMLPTFQRLSLLG--
NCU01486.1	GVGKPVVKKFERLSLLGGR
FG04361.1 	KLEPPVVTGVKKLTLLGS-
AN4413.1  	GQEPPKIKEMKKSALLS--
          	    * :  .:: :**.
```
